# Corporate Social Responsibility and Organizational Psychology: An Integrative Review

**DOI:** 10.3389/fpsyg.2016.00144

**Published:** 2016-02-16

**Authors:** Ante Glavas

**Affiliations:** Department of Corporate Social Responsibility, Strategy and Entrepreneurship, KEDGE Business SchoolMarseille, France

**Keywords:** corporate social responsibility, sustainability, organizational psychology, organizational behavior, human resources, industrial and organizational psychology (I–O psychology), corporate citizenship, social entrepreneurship

## Abstract

The author reviews the corporate social responsibility (CSR) literature that includes the individual level of analysis (referred to as micro CSR in the article) based on 166 articles, book chapters, and books. A framework is provided that integrates organizational psychology and CSR, with the purpose of highlighting synergies in order to advance scholarship and practice in both fields. The review is structured so that first, a brief overview is provided. Second, the literatures on organizational psychology and CSR are integrated. Third, gaps are outlined illuminating opportunities for future research. Finally, a research agenda is put forward that goes beyond addressing gaps and focuses on how organizational psychology and CSR can be partners in helping move both fields forward—specifically, through a humanistic research agenda rooted in positive psychology.

## Introduction

Corporate social responsibility (CSR) is an increasingly important topic for organizations. Almost every major organization is engaged to some extent in CSR. Ninety-three percent of the world’s largest companies formally report on CSR (KPMG, 2013) and it is not just limited to North America or Western Europe. For example, 69% of companies in India report on CSR, 64% in Vietnam, 60% in Philippines, and 52% in Mexico (Grant Thornton, 2013). In fact, as of 2009, more than 15% of the CSR reports in the world have originated in China (Marquis and Qian, 2014).

**FIGURE 1:**
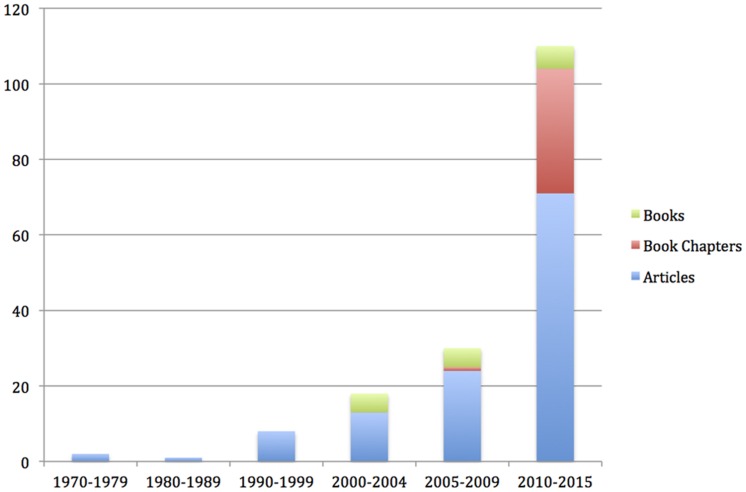
**Growth of articles, book chapters, and books in CSR at individual level of analysis**.

In parallel, the growth of the scholarly CSR literature has been exponential. In a review of the literature, Aguinis and Glavas (2012) found that over half of the peer-reviewed articles on CSR have been published in the last decade. Although most of the extant literature on CSR is at the macro (i.e., organizational) level (Lee, 2008), an increasing interest has been shown in the micro level of CSR (Aguinis and Glavas, 2012) and as seen in **Figure 1**. Moreover, in a survey of organizational psychologists conducted by the Society of Industrial and Organizational Psychology, CSR was viewed as one of the top trends affecting the workplace (Below, 2014). In addition, special issues have been published recently on the intersection of CSR and organizational psychology in leading journals such as *Personnel Psychology* (Morgeson et al., 2013), *Journal of Organizational Behavior* (Andersson et al., 2013), *Management and Organizations* (Rupp et al., 2011), and a research topic published in *Frontiers in Psychology* (Glavas et al., in review). Therefore, this review answers the calls of Aguinis (2011) and Aguinis and Glavas (2013a) to further create synergies between the fields of organizational psychology and CSR.

Despite the growing body of research on the intersection of organizational psychology and CSR (hereinafter referred to as micro CSR), there is a need for micro CSR research (Aguilera et al., 2007; Rodrigo and Arenas, 2008; Aguinis and Glavas, 2012; Rupp et al., 2013b) and thus an opportunity for involvement of organizational psychologists. As a preview of what I will outline in this manuscript, there are several gaps in the CSR literature, which also present an opportunity for new avenues of research. First, CSR has been primarily studied at the macro and institutional levels, but more studies are needed to understand how CSR influences employees. Second, even when CSR has been studied at the individual level of analysis, it has been primarily on the antecedents to employee involvement in CSR or the impact of CSR on employee outcomes (e.g., engagement, job satisfaction, organizational commitment, and organizational citizenship behaviors). Therefore, a major gap is the exploration of mediators and moderators of the CSR–employee outcome relationship. Simply put, we know that CSR has an effect on employees but we know less about why, how, and when. Third, although there are a few conceptual models that do propose more complex models of CSR (i.e., with multiple mediators and mediators), they are lacking the rigor of empirical testing, which is an area that organizational psychologists can partner with those in CSR. Fourth, there is a major gap between CSR theory and practice. While corporations are forging ahead with CSR, at the same time, they are struggling with implementing CSR—this offers an opportunity for organizational psychologists to put forward and test theory that can then be translated into models and frameworks useful for practice.

On the other hand, not only could CSR benefit from the involvement of organizational psychologists, but organizational psychologists could also greatly benefit from engaging in novel and interesting research by integrating CSR. This is especially possible if CSR in not treated as a specialty field (i.e., a separate field of study) but rather as a context within which scholars can study work in a new light (Aguinis and Glavas, 2013a). For example, scholars have found that employees are motivated by more than financial goals (Deci and Ryan, 1985; Eisenberger and Cameron, 1996), yet the field of organizational psychology has primarily focused on how employees can contribute to financial productivity and improve organizational performance (Weiss and Rupp, 2011; Hulin, 2014). As Aguinis and Glavas (2013a) stated, organizational psychology has much to offer but is often limited by those in power (i.e., management) to a focus on what produces short-term performance. The authors further propose that if organizational psychology is integrated with CSR (e.g., focus on well-being of employees and long-term performance, not just short-term performance), a more sustainable employee-employer relationship might result.

In order to integrate organizational psychology with CSR, the manuscript is structured as follows. First, I give a brief overview of CSR for those scholars not familiar with the field. The overview can even be useful for scholars who are familiar with CSR as it can improve understanding of why the field of CSR is where it is today. Second, I integrate the extant CSR literature together with organizational psychology. Finally, I put forward a research agenda for organizational psychology and CSR with a focus on potentially novel and interesting synergies.

## Brief Overview of Csr

A detailed overview of CSR is beyond the scope of this article (for reviews, see Carroll, 1999, 2008; Waddock, 2004; Aguinis and Glavas, 2012). For purposes of this review, I briefly outline a few key trends that inform why and how organizational psychology and CSR can be integrated.

### Evolution from a Focus on Institutional and Macro to a Need for the Micro Level of Analysis

First, the role of the firm has been a central debate from the beginning of CSR scholarly literature (e.g., Berle, 1931; Dodd, 1932) that has continued (Friedman, 1970; The Economist, 2005). The main question being asked, often implicitly, is whether firms have a role in society beyond economic profit.

Second, a related debate was whether CSR is normative (i.e., it is the duty of organizations to engage in CSR) and/or instrumental (i.e., it is in the interest of organizations to engage in CSR). If CSR is normative, then firms have a moral obligation to society to care for its well-being (e.g., Goodpaster, 1991; Donaldson and Preston, 1995). Other scholars have argued for an integrated view of both normative and instrumental (Swanson, 1995; Jones and Wicks, 1999).

Third, perhaps as a result of the ongoing debates, a major focus of CSR research was to avoid this conflict and to prove once and for all that CSR positively influences financial performance. Then the reasons for engaging in CSR (e.g., normative and/or instrumental) would not matter and the role of the firm would not be questioned (i.e., CSR is about doing good for both the firm and society). However, inconclusive results were found regarding the relationship between CSR and firm financial performance (Margolis and Walsh, 2003; Peloza, 2009; Wood, 2010).

These three aforementioned trends highlight the need for micro CSR research. One of the reasons for inconclusive results is that when CSR is aggregated to the macro level, the variance of both positive and negative effects on employees is not captured (Glavas and Kelley, 2014). Through micro CSR research, it is possible to unpack the results and find that CSR may under certain conditions influence some employees positively, while others negatively (Aguinis and Glavas, 2013b). Therefore, scholarship benefits by understanding these more complex findings because more holistic models of CSR can be built. Practice also benefits because firms interested in CSR can build models that enforce positive effects of CSR and minimize the negative effects.

Moreover, the debates on the role of the firm in society might be taking place at the micro level as well. CSR could be opening up questions regarding the role of work for employees (e.g., is it to secure economic profit and/or to also have a positive impact on the world). Due to the depth of these questions, it is possible that how employees perceive CSR and its importance to their own lives will vary greatly. CSR thus opens up a context within which to study numerous topics in organizational psychology such as the importance of self-concept, purpose at work, values alignment, and career development.

As a result, scholars have increasingly become more interested in micro CSR, as can be seen in **Figure 1**. Over two-thirds of the articles in micro CSR have been published in the last five years.

### Evolution of Conceptualizations of CSR

Reflecting the trends outlined in the previous section, conceptualizations of CSR have been primarily at the institutional, and macro levels (Lee, 2008). Moreover, there have been many overlapping and sometimes confusing definitions of CSR due to the various schools of thought (Carroll, 1999; Waddock, 2004). For example, in a review, Peloza (2009) found that 36 distinct measurements of CSR have been employed. Many terms have been used interchangeably with CSR such as corporate citizenship, corporate social performance, stakeholder theory, sustainability, and sustainable development to name a few. For purposes of this article, I use the definition of Glavas and Kelley (2014, p. 171) which builds on Waddock’s (2004) definition:

[CSR] is defined as caring for the well-being of others and the environment with the purpose of also creating value for the business. CSR is manifested in the strategies and operating practices that a company develops in operationalizing its relationships with and impacts on the well-being of all of its key stakeholders and the natural environment.

#### Conceptualizations of CSR

What is relevant for scholars of micro CSR is that no one definition is commonly accepted, which presents both a challenge and opportunity. The challenge is that the lack of clarity makes it difficult to generalize CSR results. This confusion also makes it even more important for scholars to precisely define what they mean by CSR in their studies. However, the opportunity is that because CSR is so broad, there is the potential for huge variance in how employees perceive CSR. For example, some employees might perceive CSR as the moral duty of a firm (e.g., care for the environment, fair wages for workers in the supply chain), while others might feel that CSR should only be used to improve relationships with key stakeholders. Then these differing perceptions could affect employee work attitudes and behaviors in varying degrees.

#### Taxonomy of CSR

In order to gain clarity for research purposes, scholars have developed different classifications of CSR, of which I outline three common categories. The first is whether CSR is focused purely on shareholder gains or if it is focused on the well-being of all stakeholders (i.e., person, group, or organization that can affect or be affected by an organization), including shareholders—referred to as sustainable value (Figge and Hahn, 2004; Laszlo, 2008). The second is whether CSR is symbolic or substantive (Meyer and Rowan, 1977)—David et al. (2007) build on the definition of Ashforth and Gibbs (1990) to define substantive as real change while symbolic change just creates the appearance of change while no actual change takes place. The third is whether CSR is peripheral or embedded in the firm—where embedded CSR is integrated into the strategy as well as daily operations (Laszlo and Zhexembayeva, 2011; Aguinis and Glavas, 2013b).

### Measurement of CSR

By combining the previous two sections (i.e., conceptualizations and taxonomies of CSR), some of the challenges for organizational psychologists in measuring CSR become evident. Most of the theoretical approaches have been at the macro level of analysis—as a result, measurements of CSR have been at the macro level as well (Glavas and Kelley, 2014). In other words, the actual perceptions of employees of their company’s CSR have not been adequately captured.

In addition, the aforementioned taxonomy of CSR informs measurement. As Aguinis and Glavas (2013b) put forward, if CSR is embedded, it inherently includes the micro level of analysis. In other words, CSR is part of the daily operations and every employee has some sort of contact with CSR. Most likely, the degree of CSR embeddedness will vary throughout the company, which then in turn means that the perception of employees of their company’s CSR will vary (Aguinis and Glavas, 2013b). That is why it is crucial to measure an employee’s perception of CSR (for a scale measuring stakeholder, substantive, and embedded CSR, see Glavas and Kelley, 2014; for a multidimensional scale see El Akremi et al., in press). Once an employee’s perception of CSR is measured, then the impact of the perceptions of CSR on employees can be measured.

Not only might employee perceptions of CSR vary, but the resulting influence of their perception on their work attitudes and behavior might vary as well. For example, some employees might perceive that if CSR is not substantive, it is then greenwashing (i.e., inauthentic) which in turn could negatively influence their perceptions of values fit with the organization. On the other hand, some employees might only care about the impact of CSR on the reputation of the organization, so for them symbolic CSR could have a positive impact on their organizational identification. Moreover, if CSR is perceived as being instrumental, some employees might be positively affected because they only care about CSR if it creates value for the company. Another possibility is that some employees might believe that CSR should only be normative (i.e., based on a moral agenda), so they will perceive that making money on CSR is hypocritical. As can be seen in these last few examples, why and how CSR impacts employees depends heavily on individual differences, what is meaningful to employees, how they construct their self-concepts, and many other individual factors—all of which are areas of organizational psychology. In sum, organizational psychology could help take CSR to a deeper level of understanding.

## Integrating CSR and Organizational Psychology

**FIGURE 2:**
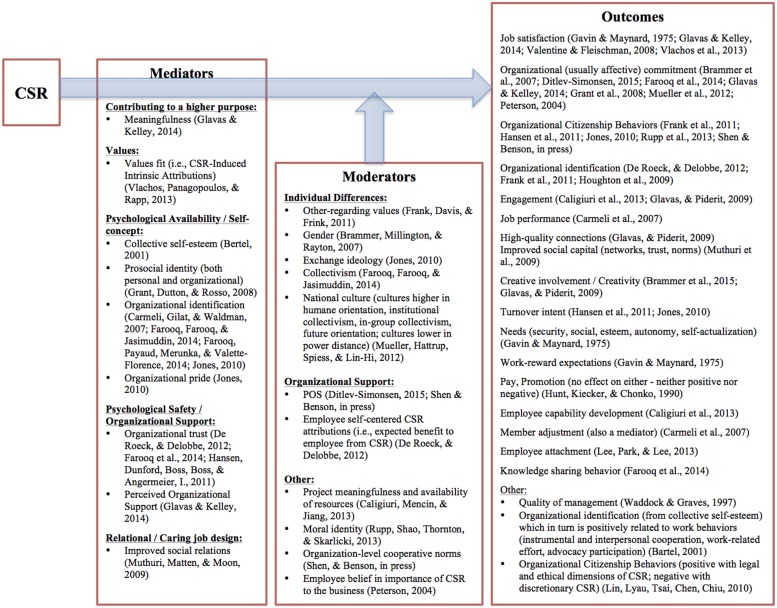
**Corporate social responsibility – outcomes relationship for incumbent employees with mediators, moderators**.

In the following section, I review the CSR literature at the individual level of analysis. Although there is no review to my knowledge on micro CSR, studies of micro CSR will often include a brief overview of the extant literature (for examples, see Aguinis and Glavas, 2013b; Rupp et al., 2013b; El Akremi et al., in press). Therefore, I try to go beyond simply summarizing the literature. Instead, I focus on integrating the extant CSR and organizational psychology literatures with the purpose being to highlight synergies that could expand our understanding of work in general. Because of length limitations, I do not cover every single article published in micro CSR. Rather I will focus on a few key themes. A more detailed overview can be found in **Figure 2**. Please note although **Figure 2** is the result of a comprehensive literature review, it is not exhaustive. Moreover, it only includes empirical research on incumbent employees—which, as will be explained later, potentially offers numerous research opportunities for organizational psychologists. The purpose of **Figure 2** is twofold. Scholars can quickly see what we know, what the gaps are, and thus envision future research that might expand the current literature. Second, **Figure 2** is also a quick guide for scholars interested in a specific domain of micro CSR, in which they can quickly get a grasp of the literature in that domain.

### Outcomes

As can be seen in **Figure 2**, CSR has numerous positive effects on employees. For example, scholars have found a positive relationship between CSR and organizational commitment (Peterson, 2004; Brammer et al., 2007; Grant et al., 2008; Mueller et al., 2012; Farooq et al., 2014a; Glavas and Kelley, 2014; Ditlev-Simonsen, 2015) as well as job satisfaction (Gavin and Maynard, 1975; Valentine and Fleischman, 2008; Vlachos et al., 2013; Glavas and Kelley, 2014).

Other positive relationships between CSR and outcomes have been found. For example CSR is positively related to organizational citizenship behaviors (OCB: Jones, 2010; Evans et al., 2011; Hansen et al., 2011; Rupp et al., 2013b; Shen and Benson, 2014). These findings suggest that if the organization goes above and beyond its primary task (i.e., financial goals) to contribute to the greater good of society (i.e., CSR), then employees will go above and beyond their primary tasks to contribute to the greater good of the organization (i.e., OCBs).

In addition, CSR is positively related to organizational identification (Houghton et al., 2009; Jones, 2010; Evans et al., 2011; De Roeck and Delobbe, 2012). These studies suggest that CSR improves an organization’s reputation which in turn leads to employees being proud to work there. Also, social identity theory would suggest that if treating others well is part of an employee’s self-concept, then they would find greater identification with an organization that treats others well (Dutton et al., 2010).

Corporate social responsibility has also been found to be positively related to high quality relationships among co-workers (Glavas and Piderit, 2009) as well as trust in relationships (Muthuri et al., 2009). These studies put forward a relational view of CSR in which CSR by its very nature includes caring for stakeholders. Therefore, it follows that organizations that put effort into creating quality relationships with external stakeholders could create a culture in which caring relationships inside the organization are important as well.

The previous outcomes are only a sampling of the potential outcomes. As shown in **Figure 2**, CSR is also positively related to other outcomes such as employee engagement (Glavas and Piderit, 2009; Caligiuri et al., 2013), creativity (Glavas and Piderit, 2009; Brammer et al., 2015), turnover intent (Jones, 2010; Hansen et al., 2011), employee attachment (Lee et al., 2013), knowledge sharing behavior (Farooq et al., 2014a), employee capability development (Caligiuri et al., 2013), quality of management (Waddock and Graves, 1997), and needs fulfillment (Gavin and Maynard, 1975).

### Underlying Mechanisms

Although the majority of CSR research at the individual level of analysis has been focused on finding a relationship between CSR and employee outcomes, there are some studies which provide insight into the underlying mechanisms. These mechanisms are important to understand because if each employee is unique in his/her own psychological reactions, then it follows that his/her reactions to CSR will most likely vary. In addition, the underlying mechanisms also provide novel insights for organizational psychologists into aspects of an employee’s work that was perhaps missing for the employee in their work (e.g., meaningfulness at work), but CSR was able to fill that gap (Glavas and Kelley, 2014).

#### Whole Self

As seen from the mediators in **Figure 2**, one of the mechanisms through which CSR influences employees is by enabling them to bring more of their whole selves to work. Kahn (1990) put forward that the more that employees are able to bring their whole selves to work, the more they will be engaged. Rich et al. (2010) further expanded on Kahn’s (1990) work and if these two studies are taken together, there are four key aspects of the whole self, which are also found among the mediators of the CSR-outcomes relationship: (a) psychological safety, (b) psychological availability, (c) values congruence, and (d) purpose.

Psychological safety helps employees show more of their whole selves at work. This is often the result of perceived organizational support, which has been found to be positively related to CSR (Glavas and Kelley, 2014). A related concept, trust in the organization, has also been found to be positively related to CSR (Hansen et al., 2011; De Roeck and Delobbe, 2012; Farooq et al., 2014b). In other words, CSR can provide nurturing and safe environments in which employees feel a safe space to show up more as who they truly are.

Second, psychological availability may stem from improved self-esteem as well as alignment of self-concept with the organization, which in turn enables employees to be more fully present at work. CSR has been found to be positively related to self-esteem (Bartel, 2001) as well as one’s self-concept (Carmeli et al., 2007; Grant et al., 2008; Jones, 2010; Farooq et al., 2014a,b). Simply put, employees might feel good about themselves by working for an organization that is doing good in the world.

A third pathway through which employees can bring more of their whole selves to work is when they feel an alignment of their values with the organization. This is the opposite of what happens when employees perceive that the values in the workplace are simply words on paper such as in a marketing brochure. However, when organizations engage in substantive CSR, employees might believe more in the values of the organization and potentially find greater values congruence (Vlachos et al., 2013). For example, employees might increase their belief in organizational values such as caring, respect, integrity if the organization is carrying out substantive CSR.

The fourth and final aspect put forward by Kahn (1990) and Rich et al. (2010) is that it is important for some employees to feel as if work contributes to a greater purpose or to the common good. The argument is that most humans have an innate desire to have a sense of purpose but often do not find this at work; therefore, they are drawn to CSR as an avenue for contributing to a greater purpose (Hulin, 2014). Although this fourth aspect might be a significant source for bringing one’s whole self to work through CSR, only one study has explored this empirically, finding a positive relationship between CSR and purpose (Glavas and Kelley, 2014).

#### Self-Interest

Employees might perceive CSR to also be of self-interest. Because CSR by definition means that organizations take care of their key stakeholders, which also includes employees, organizations high in CSR usually provide better working conditions and benefits to employees (De Roeck and Delobbe, 2012; Shen and Benson, 2014; Ditlev-Simonsen, 2015). Scholars (e.g., Swanson, 1995; Jones and Wicks, 1999) have argued that self-interest (i.e., instrumental view) is a complementary perspective to the view that CSR is only about altruism (i.e., normative view)—firms may benefit if employees perceive CSR to be of self-interest. For example, social exchange theory has been used to posit that because organizations high in CSR invest more in employees, employees will give back more (e.g., Cropanzano and Rupp, 2008). Jones (2010) found that exchange ideology moderated the relationship between employee perceived benefits of CSR and OCBs. Similarly, Rupp et al. (2006) have proposed third-party justice effects of CSR. As Rupp (2011) put forward, organizational justice has traditionally explored how an employee is treated and then responds in turn; however, in third-party justice, similar effects can be found based on how the employee perceives that the organization treats *others*. When an employee perceives that their organization treats others fairly, they will in turn expect that same fair treatment toward themselves. Because Rupp et al. (2013a) define third-party justice as CSR, this view allows for an integration of the literature on organizational justice with CSR. The same underlying mechanisms in organizational justice might also apply to micro CSR. To be clear, organizational justice theory can be integrated in other ways as well with CSR (see Rupp et al., 2006; Cropanzano and Rupp, 2008).

#### Morality

A different underlying mechanism is that CSR is simply the right thing to do. Although morality can be considered part of one’s whole self, I am listing it separately here because of the literature it comes from (i.e., ethics). For example, Rupp et al. (2013b) found that moral identity moderates the relationship between CSR and outcomes. However, despite the obvious link to the ethics literature, the literatures on ethics and CSR have largely grown in parallel although there are some conceptual studies making the bridge between the two literatures (e.g., Godfrey, 2005; Aguilera et al., 2007; Rupp, 2011; Rupp et al., 2013a).

#### Other Mechanisms

Individual differences influence how and why employees are influenced by CSR: For example, other-regarding values moderate the relationship between CSR and outcomes (Evans et al., 2011). Employee environmental values and communal orientation influence attraction to CSR (Jones et al., 2014). However, there are also counter-intuitive findings. In a meta-analysis Wiernik et al. (2013) found age to be positively related to employee involvement in CSR. This is counter to popular press which states that younger generations are more drawn to CSR (e.g., Meister, 2012; Seager, 2014). In addition, other individual differences such as gender have been found to moderate the relationship with CSR, such that females are more positively affected than males by fair working practices and the positive reputation (i.e., social responsibility) of the organization (Brammer et al., 2007). Finally, the differences between national cultures also has been explored, finding mixed results. Mueller et al. (2012) found that the relationship between CSR and employee outcomes is strengthened in cultures higher in institutional collectivism, humane orientation, in-group collectivism, future orientation, and lower in power distance. However, Farooq et al. (2014a) found that cultures high in individualism value community-related CSR. This might be a result of employees finding greater benefits from reputational effects of CSR.

### Antecedents to CSR

Although the focus of this review is not on what impacts CSR, a growing body of research been conducted on this topic. Therefore, I would be remiss in not covering this important topic because organizational psychologists have a lot to contribute to models of engaging employees in CSR. For example, theories on leadership could inform how leaders influence employees to engage in CSR (Snell, 2000). Moreover, supervisor support of CSR has been found to influence employee involvement in CSR (e.g., Weaver et al., 1999; Ramus and Steger, 2000; Hemingway and Maclagan, 2004). In addition, individual differences can also influence employee engagement in CSR such as values (Bansal, 2003; Hemingway, 2005), personality traits (Mudrack, 2007), age (Wiernik et al., 2013), and gender (Brammer et al., 2007).

For more details, numerous reviews exist on engaging employees in CSR (Aguinis, 2011; Ones and Dilchert, 2012; Norton et al., 2015). There have also been entire edited volumes (Jackson et al., 2012; Huffman and Klein, 2013) and even entire fields devoted to engagement in CSR such as humanitarian psychology and also environmental psychology (Gardner and Stern, 2002; Gifford, 2007; Koger and Du NaCnn Winter, 2010).

### Recruiting and CSR

A significant area of micro-CSR research that can inform the previous subsections on outcomes, underlying mechanisms, and antecedents of CSR is that of the relationship between CSR and firm attractiveness to prospective employees. This section is listed separately because it does not actually study incumbent employees. However, findings from the CSR recruiting literature do partially overlap with those from incumbent employees. Most of the CSR recruiting literature is guided by signaling and social identity theories, with scholars, for example, finding that CSR signals to prospective employees the values of the organization and thus the potential for values congruence (e.g., Gully et al., 2013; Jones et al., 2014). Moreover, CSR has been found to signal the organization’s reputation which then resulted in increased pride (e.g., Jones et al., 2014). Also, CSR could signal to prospective employees that they can expect to be treated fairly (e.g., Montgomery and Ramus, 2011; Jones et al., 2014).

### Summary

In sum, employees are affected by CSR through a myriad of pathways. Therefore, it is important to go beyond the simplistic direct effect of CSR-outcomes in order to understand why, how, and when employees are affected by CSR. By doing so, scholars can build more complete models of CSR. At the same time, organizational psychologists might also find valuable insight into what moves employees at work and thus expand our current theories of work.

## Gaps

The previous sections point to a few evident gaps. In the following section, the gaps are analyzed with the goal of shaping a future research agenda.

### Research on Incumbent Employees

One of the major gaps is that despite the explosion of research in micro CSR (see **Figure 1**), little is known about how employees experience CSR. Of the 166 publications that were reviewed, only 28 (or about 1/6) studied incumbent employees and their experience of CSR. Almost as many studies focused on prospective employees (18) as on incumbent employees (28). Although we can learn a lot from prospective employees (e.g., the importance of values and firm reputation), more research is needed on incumbent employees.

### Underlying Mechanisms

Even when incumbent employees have been studied, usually it has been in a mechanistic fashion, trying to prove that CSR leads to positive employee outcomes without understanding how and why. As a result, there is growing body of research, as shown in **Figure 2**, showing that CSR leads to many individual-level outcomes such as increased job satisfaction, organizational commitment, OCBs, and organizational identification. However, out of the 28 empirical studies on incumbent employees, only 11 analyzed mediators and only 12 moderators, of which only three studies explored both mediators and moderators (Jones, 2010; De Roeck and Delobbe, 2012; Farooq et al., 2014a). Moreover, only two studies of incumbent employees studied multiple mediators (Farooq et al., 2014b; Glavas and Kelley, 2014). Therefore, even when underlying mechanisms are explored, there still has been a simplistic understanding with little knowledge of which mechanisms have a greater affect on employees and under what conditions. For example, perceived organizational support has been found to influence the relationship between CSR and employee outcomes (e.g., De Roeck and Delobbe, 2012; Shen and Benson, 2014; Ditlev-Simonsen, 2015). In other words, employees are positively influenced by CSR because they perceive that CSR will benefit them directly through better work conditions and other benefits. However, these studies included perceived organizational support as the only mediator. On the other hand, Glavas and Kelley (2014) found that although perceived organizational support—when it is the only mediator—does mediate the relationship between CSR and outcomes; however, when meaningfulness is added as a mediator, the effects of perceived organizational support are negligible while meaningfulness has the strongest impact. In sum, we lack more complete models to understand why, how, and when employees are affected by CSR.

### Theory Building

In my review of the literature, I found no empirical theory-building articles on how employees might psychologically experience CSR. There are two articles which used mixed methods and the content of these articles can loosely be defined as CSR (Bartel, 2001; Grant et al., 2008) and only one which is purely qualitative and it is a case study (Muthuri et al., 2009). We are missing studies similar to those conducted on antecedents of CSR (e.g., Bansal, 2003; Rodrigo and Arenas, 2008) in which theory emerged from the data.

Simply put, CSR research is taking established models of what drives behavior in the workplace (e.g., from organizational psychology) and testing them out on CSR. In fact, 90% of the articles on incumbent employees are quantitative and most of them empirically test models through surveys. Although quantitative studies do provide insight, additional insight could be gained by inductively studying why, how, and when CSR affects employees. We may be surprised to find that CSR opens up new ways of looking at our models of work.

### CSR as a Field in and of Itself

Corporate social responsibility has been a field that has been fairly closed off and separate (Aguinis and Glavas, 2012), which might be why organizational psychologists have not been more involved. Over half of the publications (94) explored antecedents of CSR action (i.e., how to drive employee involvement in CSR). Moreover, in the 28 studies that explored how CSR affected incumbent employees, less than half of the studies built on any theory outside of CSR. In other words, studies were conducted to show the direct effect of CSR on outcomes. In sum, CSR often has not integrated other fields; yet, it is a context within which multiple disciplines can be applied (e.g., organizational psychology).

### Other Gaps

Numerous other gaps exist such as that studies are lacking from outside of North America and Western Europe as well as studies of small and medium enterprises. Moreover, two gaps seem to especially stand out. First, studies on incumbent employees are not bridging practice and scholarship (cf. Aguinis, 2011). As mentioned previously, preconceived models are tested on employees, but there is little theory building (e.g., grounded theory). This trend in micro CSR is similar to the trend in the broader CSR literature that Waddock (2004) observed which is that academia and practice exist in parallel universes. In other words, scholars are not going out into the workplace to truly investigate why, how, and when employees experience CSR. Second, multilevel models are needed. This is an extremely important point, also brought up by Aguinis and Glavas (2012), because in the push for micro CSR, scholars should be weary that the micro-macro divide is not further increased.

### Mechanistic Approach

With the focus on trying to show that CSR has an impact on employees, it seems that the actual human being has been overlooked. By taking a look at **Figure 2**, the extant research seems very mechanistic, with arrows drawn from antecedents to outcomes. To be clear, I am not implying that organizational performance is not crucial for organizations, but rather that we need more studies on the actual human experience of CSR (e.g., how and why CSR affects employees).

As will be shown in the following section on future research, all the gaps mentioned in this section are opportunities for future research. As scholars in organizational psychology have put forward, our workplaces have become too mechanistic (Weiss and Rupp, 2011; Hulin, 2014) and CSR could be a major opportunity for organizational psychologists to study how to contribute to a more humanistic view of work (cf. Pirson and Lawrence, 2010).

## A (Humanistic) CSR Research Agenda

As shown in **Table 1**, the previously identified gaps lead to a rather straightforward research agenda for organizational psychology and CSR. Specifically, the gaps point to a need for future micro CSR research to (a) focus more on incumbent employees, (b) explore underlying mechanisms especially in more complex models such as with multiple mediators and moderators, (c) create theory that comes from the actual phenomena (e.g., grounded theory), and (d) include multiple disciplines with a focus on CSR that can also contribute back to the theory in those disciplines.

**Table 1:** Future research opportunities and synergies for CSR and organizational psychology.

Topic	Potential Research Questions
Whole/ideal self	• All things held equal, how, when, and why can CSR lead to employees living out more of their whole and ideal selves at work?
	• How can organizations use CSR as an employee engagement strategy by enabling employees to bring more of their whole selves at work?
Meaningfulness	• For whom is finding meaningfulness important at work and how can CSR be a source for meaningfulness at and in work?
	• How can organizations design performance management systems that go beyond pay and promotion to also include if employees are carrying out work that is meaningful for them, the organization, and society? Moreover, how can CSR be a pathway for such multilevel models of meaningfulness (to the individual, organization, society)?
Job design	• Can CSR be used as a means for creating relational job designs?
	• Is it possible to create caring and compassionate cultures through CSR? If so, how? And under what conditions are employees positively and/or negatively affected by caring and compassionate cultures?
Creative potential	• Why, how, and when does CSR lead to the unleashing of creative potential?
Other underlying mechanisms	• What are other mediators and moderators that influence the relationship between CSR and employee outcomes?
Methodology	• We need more qualitative studies (e.g., grounded theory) that actually uncover theories of why employees are attracted to CSR. Then these theories can be refined into models relevant for organizational psychology.
	• We also need to be careful that we do not create another silo of research with CSR at the individual level. More multilevel studies are needed.
	• Action research is needed in which we research what is possible for CSR and organizational psychology. Scholars can be at the forefront rather than collecting past data and only making incremental contributions.
	• Models with multiple mediators and moderators are needed in order to create more comprehensive models and avoid false positive findings of more simplistic models.
Practice	• Although all the above opportunities have related questions for practice, an underlying stream relevant for practice is how can organizational psychologists, with their great capability to create models and systems, create ones for implementation of CSR that also improve the workplace?


As seen in **Figure 2**, there are many approaches that can be taken to CSR—in other words, many areas of organizational psychology and management in general are connected to CSR. As Morgeson et al. (2010) stated, despite a century of research, we will still lack an understanding of how the broader organizational context impacts work. If the broader context is assumed to be society and the environment, then CSR is a perfect conduit to understanding how context impacts work (Aguinis and Glavas, 2013a). Therefore, not only can scholars from other disciplines help CSR, but CSR can also help scholars from other disciplines test out novel and interesting models within the context of CSR (Aguinis and Glavas, 2013b).

Moving beyond the more straightforward needs for future research evident from the Gaps section, in the following section I further integrate organizational psychology and CSR to propose a few additional research topics. In sum, the first part of this article dealt with what we know regarding the synergy between organizational psychology and CSR, the second part dealt with what we do not know, and this following section deals with some possibilities of what we could know.

### Whole and Ideal Self

As mentioned previously, we know from engagement theory (Kahn, 1990; Rich et al., 2010) that the more that employees bring of their whole selves to work, the more they will be engaged. Yet at the same time, it seems that our workplaces are designed so that we only bring part of our whole selves to work. As a result, less than 30% of employees are engaged at work (Gallup, 2013) and work is not one of the top eight reasons that makes people happy (Wallis, 2005). This is even more troubling from a well-being perspective because our lives are increasingly revolving around work (Rosso et al., 2010; Hulin, 2014).

Specifically, CSR could be a conduit for bringing more of the whole self to work (Kahn, 1990; Rich et al., 2010) through the psychological availability to show up whole, an alignment with the values of the organization, and/or being able to contribute to a higher purpose. As shown in **Figure 2** and also outlined in the review section on underlying mechanisms, CSR could enable all four of these factors. In summary, future research could explore if CSR is a conduit for people to show up whole at work. Perhaps this is why some employees might be attracted to CSR.

As a related topic, CSR is therefore a context within which employees can live out their ideal self, which is one’s purpose, passion, and values (Goleman et al., 2001). In contrast, the ought self is what one feels that he/she is obligated to do based on societal conditioning and external pressure (Boyatzis and McKee, 2005). Often employees live out their ought selves at work for reasons such as being conditioned to believe that they should be in a certain profession and/or act in a specific manner at work (Boyatzis and McKee, 2005).

### Meaningfulness

Future research could also focus on theories and empirical studies of CSR and meaningfulness. Management systems are often designed to motivate employees based on pay and promotion, but overlook important needs such as meaningfulness (Wrzesniewski, 2003). One way that employees find meaning is by contributing to the common good or CSR (Rosso et al., 2010). However, we still know little about the actual process of why and how employees could find meaningfulness through CSR.

### Relational Job Design

As scholars (Glavas and Kelley, 2014; Aguinis and Glavas, in review) put forward, CSR could expand current job design theory to be also relational. Grant (2007) stated that our job design literature has mostly stagnated in the last couple of decades. Moreover, Grant (2008a) put forward that models of job design could be expanded so that significance is not only constrained to one’s task but rather that one’s job in general could be significant and meaningful. Grant (2008b) found support that employees are positively impacted when they engage in prosocial behaviors and especially when they see that they have improved the well-being of beneficiaries with whom they have contact (e.g., stakeholders). As a result, Grant (2007) called for a relational model of job design that is prosocial in nature, which then results in greater significance and meaning in one’s job. Because CSR is prosocial in nature and is relational (i.e., caring for stakeholders), CSR offers an opportunity to expand the nature of job design to one that is relational.

By using CSR as a way to create a relational job design, it also opens up the door for creating cultures that are caring and compassionate (Aguinis and Glavas, in review). As the authors state, scholars recently (e.g., Rynes et al., 2012) have called for the study of caring and compassionate cultures in order to overcome the predominant focus of management on cultures that are rooted more in aggressiveness, competitiveness, and rigid norms. Because of the relational nature of CSR, future research could explore how creating caring relationships (i.e., caring for well-being of stakeholders) has an impact on employees. It is well-known that many employees do not thrive in cut-throat cultures and that a glass ceiling effect keeps those with more nurturing values from being fully engaged in such cultures (Van Vianen and Fischer, 2002). Therefore, CSR could be a bridge with the diversity and other related literatures on how to create workforces that engage more of our employees.

### Creative Potential

For the sake of clarity, creativity and creative potential are two different constructs. Simply put, creativity is one’s ability to approach problems and solutions and then come up with new ideas, while creative potential is how much an employee taps into that ability (Amabile, 1998). What is relevant for CSR is how much one is using their creative potential. Findings from past research suggest that influencing creativity can be quite difficult (Vernon, 1989). On the other hand creative potential can be influenced (Amabile, 1998).

Future research could focus on how CSR can be a driver for employees to use more of their creative potential. As findings from Glavas and Piderit (2009) suggest, CSR could be positively related to creative potential, but we know less about why, how, or when. One possibility is that CSR is a topic that employees feel passionate about. As Amabile (1998) put forward, passion is one of the key drivers of creative potential. When employees care about an issue (e.g., CSR), they will spend their free moments thinking about potential innovation.

### Action Research

There is a parallel situation taking place in CSR practice from which scholars could learn. Corporations are learning that disruptive innovation will not come from making incremental shifts based on old decision-making models (Brown, 2009). The challenge is that when decisions are made based solely on past information (e.g., market, financial information) and then a gap analysis is conducted, corporations are then stuck in the same mental models, which then usually leads to incremental improvements. Rather, areas such as design thinking (Brown, 2008, 2009) are teaching corporations to work from the question of what is possible. In other words, the starting point is the future and not the past.

In academia, we analyze past information and often publish at least a few years after something has taken place—that is assuming the research was even conducted based on what is going on in practice. In addition, scholars often conduct a gap analysis of the literature in order to see where a contribution can be made. Thus, there is a risk of being stuck in a perpetual loop of building on old models based on old information.

CSR offers an opportunity to break out and ask what is possible. It is the questions that we ask that define our intent and our work. One such path to conduct research is action research (Reason and Bradbury, 2001). Instead of lagging behind practice, we as scholars could be putting forward models that we then test out in practice, refine, publish, test again, and so forth. This would not only be useful for corporations, but also to researchers because access to samples would probably be easier if corporations found benefit in the work.

## Conclusion

Ironically, CSR is about caring for humans and the planet, yet in the quest to prove that CSR matters (i.e., mechanistic focus on antecedents and outcomes), we have forgotten the actual human being. As Weiss and Rupp (2011) put forward, organizational psychologists have been so focused on what leads to performance that they have also ignored the actual human being (Aguinis and Glavas, in review). Moreover, Weiss and Rupp (2011, pp. 94–95) make an analogy to a television show: “*Ice Road Truckers* is about people driving their trucks. I–O psychology seems mostly to be about whether their legs are long enough to reach the pedals.” The focus has been so much on what is good for the organization, that we have overlooked the actual driver, the engine, and the fuel.

## Author Contributions

The author confirms being the sole contributor of this work and approved it for publication.

## Conflict of Interest Statement

The author declares that the research was conducted in the absence of any commercial or financial relationships that could be construed as a potential conflict of interest.
